# The Association between Habitual Green Tea Consumption and Comprehensive Frailty as Assessed by Kihon Checklist Indexes among an Older Japanese Population

**DOI:** 10.3390/nu13114149

**Published:** 2021-11-19

**Authors:** Hinako Nanri, Tsukasa Yoshida, Yuya Watanabe, Hiroyuki Fujita, Misaka Kimura, Yosuke Yamada, on behalf of the Kameoka Study Group

**Affiliations:** 1 National Institutes of Biomedical Innovation, Health and Nutrition, Tokyo 162-8636, Japan; t-yoshida@nibiohn.go.jp (T.Y.); yu15-watanabe@my-zaidan.or.jp (Y.W.); yamaday@nibiohn.go.jp (Y.Y.); 2 Institute for Active Health, Kyoto University of Advanced Science, Kyoto 621-8555, Japan; fujita.hiroyuki@kuas.ac.jp (H.F.); kimura.misaka@kuas.ac.jp (M.K.); 3 Physical Fitness Research Institute, Meiji Yasuda Life Foundation of Health and Welfare, Tokyo 192-0001, Japan

**Keywords:** green tea, comprehensive frailty, Kihon Checklist

## Abstract

Background: It is unclear whether habitual green tea consumption is related to comprehensive frailty. Objectives: We conducted this study to investigate this relationship among an elderly Japanese population. Methods: This was a cross-sectional study of baseline data from 2012. The study included 5668 Japanese participants (2766 men and 2902 women aged 65 years or older). The subjects completed a validated self-administered food frequency questionnaire that included questions on their green tea consumption. We evaluated comprehensive frailty using a 25-item Kihon Checklist (KCL), which comprised seven domains (instrumental activities of daily living (IADL), physical function, malnutrition, oral or eating function, socialization and housebound, cognitive function, and depression). Frailty was defined as a KCL score greater than or equal to seven. Results: We found that a higher consumption of green tea was associated with a lower prevalence of comprehensive frailty in both sexes. Further age-stratified analysis showed that a higher consumption of green tea among women was associated with a lower prevalence of comprehensive frailty, regardless of age. In men, however, this association was found only in the older age groups. An analysis of the association between green tea consumption and the frailty subdomains showed that green tea consumption was associated with a lower prevalence of oral dysfunction and cognitive problems in both sexes. In addition, only in women was higher green tea consumption found to be associated with a lower prevalence of IADL and mobility-related disability problems. Conclusions: Green tea consumption is inversely associated with the prevalence of comprehensive frailty in Japanese men and women. Longitudinal studies are required to confirm this association.

## 1. Introduction

Tea, one of the most commonly consumed beverages worldwide, has beneficial effects on human health [1]. Green tea in particular contains more polyphenols, such as catechins (belonging to the flavonoid group), than other types of tea (e.g., black and oolong tea) made from the same plant (*Camellia sinensis*) [2]. Catechins, especially (-)-epigallocatechin-3-gallate (EGCG), are known to act as antioxidants in vitro; they prevent oxidation or increase the cellular lipid antioxidant activity of vitamins C and E in humans [2,3]. Numerous epidemiologic studies have reported an inverse association of green tea consumption with the risk of chronic inflammatory diseases such as type 2 diabetes mellitus, cancer, and depression [4,5,6]. Additionally, in animal and observational studies, habitual green tea consumption has been reported to have beneficial effects on age-related disorders [7,8].

Frailty is an age-related physiological syndrome, and it increases the incidence of health outcomes such as falls, hospitalization, and death; therefore, early prevention is critical [9,10]. Although there is still some debate regarding the definition of frailty, the phenotype of physical frailty, as defined by Fried and colleagues, is characterized by unintentional weight loss, global muscle weakness, exhaustion or poor endurance, slowed performance, and low levels of physical activity [9]. More recently, frailty has been considered to be multifaceted, including components such as physical frailty as well as psychological and sociological frailty [11,12]. There is growing evidence on the association between frailty and chronic inflammation [13]. Pro-inflammatory cytokines may influence frailty either directly, by promoting protein degradation, or indirectly by affecting important metabolic pathways [14]. In addition, several epidemiological studies have reported the important role of dietary factors in preventing frailty [15,16].

Several studies have reported inverse associations of frailty risk with food and nutrient intake [17,18,19,20]. A meta-analysis of four cohort studies reported that high adherence to a Mediterranean diet, characterized by a high intake of antioxidant-rich fruits and vegetables, is associated with a significantly lower physical frailty risk [17]. Previous studies have shown that antioxidants (i.e., vitamin C, vitamin E, and polyphenols) or dietary non-enzymatic antioxidant capacity were inversely associated with physical frailty risk [18,19,20]. However, it is unclear whether the habitual consumption of green tea, which contains high levels of antioxidants, is associated with comprehensive frailty. Furthermore, given that people with oxidative stress-related risk factors have a higher bioavailability of antioxidants than those without [21], the effect of green tea consumption on frailty may be greater in postmenopausal women, who are at a higher risk of chronic inflammation, than in men. The aim of the present study was to investigate the association between habitual green tea consumption and frailty by sex among an elderly Japanese population. We also examined the relationship between green tea intake and the seven subdomains that constitute comprehensive frailty.

## 2. Methods

### 2.1. Study Subjects

We analyzed baseline data from the population-based Kyoto–Kameoka Study, which aimed to examine the associations between food intake, nutritional status, physical activity, oral function, and long-term care (LTC) insurance among community-dwelling older people in Kyoto prefecture, Japan [22]. The study participants and methods used in the Kyoto–Kameoka Study have been described in detail elsewhere [22]. The Daily Life Area Needs Survey was conducted by mail among 18,231 elderly people aged 65 years or older living in Kameoka City, Kyoto Prefecture (as of 1 July 2011), excluding those who required nursing care at level 3 or higher, and 13,159 of them responded (response rate: 73.2%). Then, after excluding 69 people who died and those who required support or care, an additional survey of 12,054 people was conducted in February 2012, and the number of respondents was 8319 (response rate: 69.4%).

From the 8319 participants, we further excluded 2681 who met any of the following conditions: missing data on the consumption of green tea (*n* = 375) or the Kihon Checklist score (*n* = 2276). Finally, 5668 participants were found to be eligible for this study (2766 men and 2902 women). We described the study concept in the mail survey. A response to and return of the questionnaire with the participant’s name was regarded as them giving consent to participate in this study. The entire study protocol was reviewed and approved by the Ethics Committees of the Kyoto Prefectural University of Medicine (RBMR-E-363), the Kyoto University of Advanced Science (No. 20-1), and the National Institutes of Biomedical Innovation, Health and Nutrition (NIBIOHN-76-2).

### 2.2. Measurements

#### 2.2.1. Dietary Assessment

Dietary intake was assessed using a commonly used previously validated Japanese food frequency questionnaire (FFQ) [23,24,25,26]. In this FFQ, we asked participants to report their intake frequency to assess the average intakes of 46 food and beverage (green tea and coffee) items over the past year. The frequencies of green tea consumption were categorized as follows: almost none, 1 to 3 times/month, 1 to 2 times/week, 3 to 4 times/week, 5 to 6 times/week, 1 time/day, 2 times/day, and ≥3 times/day. The total energy intake was calculated using a program developed by Tokudome et al. [23,24] based on the standard tables of food consumption in Japan (fifth revised edition) [27].

#### 2.2.2. Definition of Frailty

In Japan, the Kihon Checklist (KCL) was developed by the Japanese Ministry of Health, Labor, and Welfare to screen for future risks of LTC certification [28,29]. Recently, the KCL was validated for use in screening community-dwelling older residents for frailty [28]. It was translated to other languages and used in various countries [30]. The strengths of the KCL include its use in the assessment of physical, sociological, and psychological domains as a comprehensive questionnaire: (1) questions 1–5 assess “instrumental activities of daily living (IADL)”, (2) questions 6–10 assess “physical function/strength”, (3) questions 11–12 assess “malnutrition”, (4) questions 13–15 assess “oral function/eating”, (5) questions 16–17 assess “socialization/housebound”, (6) questions 18–20 assess cognitive/memory, and (7) questions 21–25 assess “depression/mood” [29]. When the dichotomous response (yes/no) indicates a risk of frailty, +1 is added to the KCL score. A high KCL score (range 0–25) indicates worse functioning and severe frailty. A total KCL score of ≥7 points indicates a higher risk of long-term care [28]. The cut-off points at which problems were defined for the following seven domains were as follows: (1) IADL score of ≥3 points, (2) mobility disability score of ≥3 points, (3) nutrition score of 2 points, (4) oral or eating function score of ≥2 points, (5) socialization and housebound problems were indicated in people who answered ‘no’ to Q16 (‘Do you go out at least once a week?’), (6) cognitive function score of ≥1 point, and (7) depression score of ≥2 points [28].

#### 2.2.3. Other Variables

A self-administered questionnaire was used to assess height, weight, alcohol consumption status, and smoking status. Alcohol consumption status was classified into the following categories: everyday, sometimes, seldom, and never. Smoking status was classified into the following categories: everyday, sometimes, formerly, and never. BMI was calculated as the self-reported body weight (in kg) divided by the square of the self-reported height (in m^2^).

### 2.3. Statistical Analysis

All statistical analyses were performed separately for men and women with the SAS statistical software package (Ver. 9.4 for Windows; SAS Institute, Cary, NC, USA). We categorized their green tea consumption based on their beverages consumption distribution as follows: almost none, <1 cup/day, 1–2 cups/day, ≥3 cups/day. The participants’ characteristics according to categories of green tea consumption were compared by linear regression analysis (for continuous variables) or the Mantel–Haenszel test (for categorical variables), as appropriate. Adjusted odds ratios (ORs) and 95% confidence intervals (CIs) for frailty risk according to the consumption of green tea were estimated using logistic regression analysis. Adjustments were made for age (years, continuous), BMI (kg/m^2^, continuous), total energy intake (kcal/day, continuous), alcohol consumption status (everyday, sometimes, seldom, or never), and smoking status (everyday, sometimes, formerly, or never). We found that subjects with a higher consumption of green tea were more likely to consume coffee. We further adjusted for coffee consumption (almost none, <1 cup/day, 1 cup/day, or ≥2 cups/day) in each model as a covariate. A *p* value < 0.05 was considered statistically significant.

## 3. Results

Table 1 shows the characteristics of the participants stratified by sex. The proportion of current drinkers or current smokers and the mean total energy intake were higher among men than among women, whereas the average age, proportion of those who were underweight, and the intake frequency of fruits and vegetables were higher among women than among men. The mean total KCL score was 5.0 for men and 5.1 for women, and the prevalence of KCL frailty risk (total KCL score ≥7) was 29.3% in men and 30.6% in women, which were not statistically significant sex differences. The proportion of those with IADL and cognitive and memory problems was higher among men than among women, whereas the proportion of those with physical function/strength problems, malnutrition problems, or socialization and housebound problems was higher among women than among men.

Table 2 shows the basic characteristics of the study participants according to their consumption of green tea. Men who consumed more green tea tended to be older, have a higher total energy intake, have a more frequent fruit and vegetable intake, and have a higher coffee consumption. In addition, a lower proportion of these men tended to be current smokers. Women who consumed more green tea tended to be younger, and other factors were similar to the associations for men.

Table 3 shows multivariate-adjusted ORs and 95% Cis for comprehensive frailty according to subjects’ green tea consumption. A higher green tea consumption was associated with a lower prevalence of comprehensive frailty in both sexes (*p* for trend = 0.02 in men and <0.01 in women). Men who consumed green tea at a rate of three or more cups per day had a lower prevalence of frailty than those who had almost none (OR = 0.71, 95% CI = 0.54–0.94). Among women, there were significantly lower odds of frailty in all green tea consumption groups compared to those who had almost none (for <1 cup/day, OR = 0.67, 95% CI = 0.49–0.92; for 1–2 cups/day, OR = 0.51, 95% CI = 0.37–0.70; and for ≥3 cups/day, OR = 0.60, 95% CI = 0.44–0.81). In an age-stratified analysis, among men, the *p* value for the interaction between green tea consumption and age was 0.01. A significant trend association between green tea consumption and comprehensive frailty was observed among those in the ≥75 years age group (*p* for trend <0.01) in men; a green tea consumption of ≥3 cups per day was associated with a lower prevalence of comprehensive frailty, compared with the consumption of almost none (OR = 0.46, 95% CI = 0.30–0.72). Meanwhile, there was no such association for those in the <75 years age group (*p* for trend = 0.57). In women, there were no significant interactions between green tea consumption and age (*p* for interaction = 0.15). In the group aged <75 years, the prevalence of comprehensive frailty was significantly reduced in all consumption groups compared with those who had almost none (<1 cup/day, OR = 0.66, 95% CI = 0.44–0.99; 1–2 cups/day, OR = 0.41, 95% CI = 0.27–0.62; and ≥3 cups/day, OR = 0.51, 95% CI = 0.35–0.75). In women aged 75 years and older, habitual green tea consumption statistically significantly decreased the odds of comprehensive frailty compared with the consumption of almost none (1–2 cups/day, OR = 0.60, 95% CI = 0.37–0.995; ≥3 cups/day, OR = 0.61, 95% CI = 0.38–0.97).

**Figure 1:**
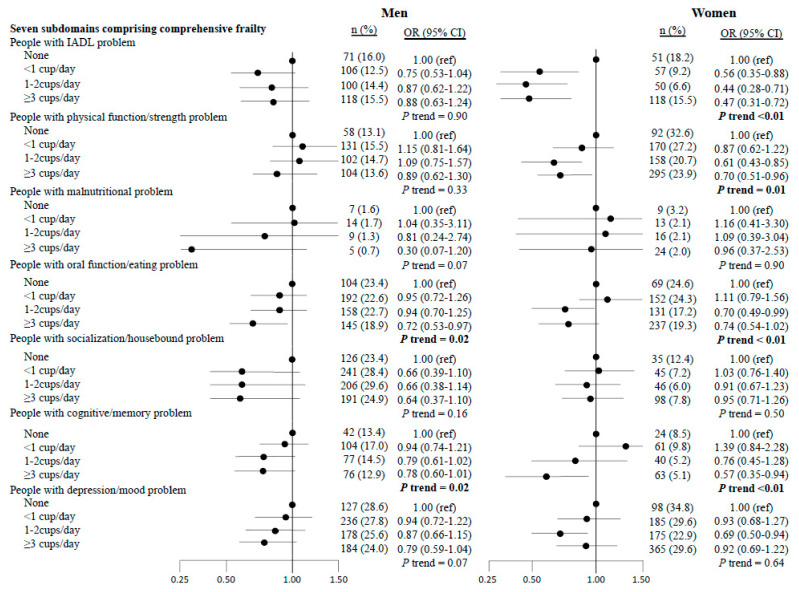
Multivariate-adjusted odds ratios (OR) and 95% confidence intervals (CI) of the seven subdomains that constitute comprehensive frailty according to green tea consumption. Adjusted for age (years), body mass index (kg/m^2^), total energy intake (kcal/day), alcohol status (everyday, sometimes, seldom, or never), smoking status (everyday, sometimes, former, or never), vegetable intake, fruit intake, and coffee consumption (almost none, <1 cup/day, 1–2 cups/day, ≥3 cups/day).

The results for the seven subdomain risks (Figure 1) showed a significant inverse association between green tea consumption and oral dysfunction (*p* for trend = 0.02 in men and *p* < 0.01 in women) and cognitive problems (*p* for trend = 0.02 in men and *p* < 0.01 in women) in both sexes. In women only, there were inverse associations between green tea consumption and IADL (*p* for trend <0.01) and mobility-related disability problems (*p* for trend = 0.01).

## 4. Discussion

In a large population-based cohort study of older adults, we found that the prevalence of comprehensive frailty was lower with the consumption of 1–2 cups of green tea per day in women and 3 or more cups per day in men. Further age-stratified analysis showed that women who consumed more green tea had a lower prevalence of comprehensive frailty, regardless of age. In men, however, this association was found only in the older age groups. An analysis of the association between green tea consumption and comprehensive frailty subdomains showed that, among both men and women, green tea consumption was associated with a lower prevalence of oral dysfunction and cognitive problems. In addition, only in women, a higher green tea consumption was inversely associated with a lower prevalence of IADL and mobility-related disability problems. To our knowledge, this is the first observational study of the association between green tea consumption and comprehensive frailty risk. 

Although there are currently no studies with which we can directly compare our results, our findings agree with those from previous reports on associations between antioxidant nutrient intake and frailty risk [18,19,20]. The Invecchiare in Chianti study reported that participants in the lowest quintile of vitamin C intake (OR, 2.12; 95% CI, 1.34–3.36; *p* for trend = 0.001) and vitamin E intake (OR, 1.96; 95% CI, 1.25–3.07; *p* for trend = 0.004) had an increased risk of frailty compared with those in the highest quintile [18]. Rabassa et al., in a longitudinal study, found that high habitual dietary resveratrol exposure was associated with a lower risk of frailty [19]. Moreover, Kobayashi et al. reported that a higher dietary non-enzymatic antioxidant capacity was inversely associated with frailty risk [20]. 

In the present study, in women a higher consumption of green tea was associated with a lower prevalence of comprehensive frailty, regardless of age. It has previously been reported that a decrease in sex hormones in postmenopausal women increases the risk of chronic inflammation due to increased levels of C-reactive protein and interleukin-6 [21]. Chronic inflammation has been shown to promote proteolysis and muscle catabolism, which in turn promotes increased risk of frailty [31]. In fact, the previous study showed that women become frail at a younger age than men [9]. We suggest that the habitual intake of green tea, with its strong antioxidizing effect, may prevent frailty in women from an early stage. 

Although the mechanism of the beneficial effect of green tea consumption on comprehensive frailty remains unclear, there are possible biological explanations. Green tea polyphenols, mainly catechins, have been shown to protect humans against oxidative stress and inflammation, which can cause muscle atrophy and muscle fiber loss [32]. EGCG have also been shown to increase antioxidant activity in animal models, and thus enhance the overall chemo-preventative effect of antioxidants in those cells and tissues [33]. Additionally, green tea consumption can have a beneficial effect on oral health [34,35]. An in vivo study suggested that green tea catechins inhibit the growth of *P. gingivalis* [34]. In addition, green tea consumption was associated with a higher general oral health assessment index [7]. Maintaining good oral health can help prevent malnutrition, weight loss, and a decline in social activities [36]. Moreover, among the Japanese population, green tea is considered an important source of certain nutrients, such as antioxidant vitamins and polyphenols, but it is also a main constituent of beverage intake [37,38]. An observational study suggests that good hydration status prevents the risk of cognitive function decline [39]. Moreover, a higher consumption of water is positively associated with a decline in oral-related quality of life [40]. These results support the findings of our data that there is an association between green tea consumption and the frailty subdomains.

Our study had some limitations. First, because the present study was cross-sectional, we were unable to conclude that the observed associations were causal. Second, the assessments of green tea consumption and comprehensive frailty were based on a self-administered questionnaire, which raises the problem of potential misclassification. Third, despite making adjustments for the main potential confounders, the possibility of residual confounding cannot be ruled out. Fourth, the number of nutrition problems, one of the components of comprehensive frailty, was relatively small. This could lead to our study having a low statistical power in detecting significant associations. Finally, the present study was conducted only among a Japanese population. The ingredients of Japanese green tea could differ from green tea types in other countries. Therefore, the findings of this study may not apply to other populations.

## 5. Conclusions

The present study confirmed that green tea consumption is inversely associated with the prevalence of comprehensive frailty. Future longitudinal studies are required to confirm that habitual green tea consumption can predict the future development of frailty.

## Figures and Tables

**Table 1:** Characteristics of the study subjects for men and women.

	Men	Women	*p* Value *
	(*n* = 2766)	(*n* = 2902)	
Age (years)	73.1 (5.7) ^†^	73.5 (6.0)	0.01
<75 years	1798 (65.0)	1828 (63.0)	0.11
≥75 years	968 (35.0)	1074 (37.0)	
Body mass index (kg/m^2^)	23.1 (2.7)	22.5 (3.3)	<0.001
Underweight, <18.5	109 (4.0)	266 (9.2)	<0.001
Normal weight, 18.5–24.9	2040 (74.2)	2053 (71.3)	
Overweight, ≥25.0	602 (21.9)	562 (19.5)	
Current drinker, *n* (%)			
Everyday	1407 (51.1)	178 (6.2)	<0.001
Sometimes	456 (16.6)	431 (14.9)	
Almost never drink	509 (18.5)	819 (28.3)	
Never	380 (13.8)	1462 (50.6)	
Current smoker, *n* (%)			
Everyday	459 (16.6)	81 (2.8)	<0.001
Sometimes	69 (2.5)	18 (0.6)	
Almost never smoke	1655 (60.0)	199 (6.9)	
Never	577 (21.0)	2574 (89.6)	
Total energy intake (kcal/day)	1915 (525)	1577 (333)	<0.001
Fruit intake (times/day)	0.79 (0.79)	1.11 (0.92)	<0.001
Vegetable intake (times/day)	3.28 (2.28)	4.11 (2.65)	<0.001
Coffee consumption ≥1 cup/day, *n* (%)	1008 (35.0)	1876 (65.1)	0.025
Total KCL score (range: 0–25)	5.0 (4.1)	5.1 (4.3)	0.09
Comprehensive frailty based on KCL score	810 (29.3)	889 (30.6)	0.27
KCL subdomains			
People with IADL problems	396 (14.3)	270 (9.3)	<0.001
People with physical function/strength problems	398 (14.4)	715 (24.6)	<0.001
People with malnutrition problems	35 (1.3)	62 (2.1)	0.01
People with oral function/eating problems	603 (21.8)	589 (20.3)	0.16
People with socialization/housebound problems ^‡^	133 (4.8)	222 (7.7)	<0.001
People with cognitive/memory problems	254 (9.2)	188 (6.5)	<0.001
People with depression/mood problems	730 (26.3)	823 (28.4)	0.10

KCL, Kihon Checklist; IADL, Instrumental activities of daily living. * *p* values for sex difference are based on *t* test for continuous variables and chi-square tests for categorical variables. ^†^ Values are mean (standard deviation) for continuous variables and number (percentage) for categorical variables. ^‡^ People who answer ‘no’ at Q16 have socialization/housebound problems.

**Table 2:** Characteristics of the study subjects according to categories of green tea consumption.

	Green Tea Consumption				*p* for Trend *
	None	<1 Cup/Day	1–2 Cups/Day	≥3 Cups/Day	
Men, *n* (%)	445 (16.1) ^†^	850 (30.7)	699 (25.3)	772 (27.9)	
Age (years)	72.4 (5.4)	72.3 (5.3)	73.5 (5.8)	74.0 (6.0)	<0.001
Body mass index (kg/m^2^)	23.1 (2.7)	23.2 (2.8)	23.3 (3.0)	23.0 (2.7)	0.34
Current drinker, *n* (%)	307 (69.3)	579 (68.5)	475 (68.2)	502 (65.4)	0.054
Current smoker, *n* (%)	110 (24.7)	176 (20.7)	121 (17.4)	121 (15.7)	<0.001
Total energy intake (kcal/day)	1833 (573)	1885 (538)	1899 (498)	2010 (492)	<0.001
Fruit intake (times/day)	0.63 (0.75)	0.68 (0.65)	0.85 (0.75)	0.95 (0.95)	<0.001
Vegetable intake (times/day)	2.76 (1.99)	2.84 (1.73)	3.39 (2.05)	3.98 (2.89)	<0.001
Coffee consumption ≥ 1 cup/day, *n* (%)	259 (58.3)	448 (52.8)	520 (74.7)	486 (63.4)	<0.001
Women, *n* (%)	282 (9.7)	625 (21.5)	763 (26.3)	1232 (42.6)	
Age (years)	74.0 (6.6)	73.1 (5.8)	73.0 (5.8)	73.8 (6.1)	0.006
Body mass index (kg/m^2^)	22.6 (3.7)	22.6 (3.3)	22.5 (3.2)	22.3 (3.3)	0.34
Current drinker, *n* (%)	52 (18.5)	133 (21.5)	182 (23.9)	242 (19.7)	0.51
Current smoker, *n* (%)	13 (4.7)	32 (5.2)	23 (3.0)	31 (2.5)	<0.001
Total energy intake (kcal/day)	1498 (387)	1518 (331)	1552 (310)	1638 (320)	<0.001
Fruit intake (times/day)	0.89 (0.93)	0.90 (0.73)	1.14 (0.84)	1.24 (1.02)	<0.001
Vegetable intake (times/day)	3.48 (2.97)	3.58 (2.09)	4.07 (2.21)	4.54 (2.97)	<0.001
Coffee consumption ≥1 cup/day, *n* (%)	146 (52.1)	345 (55.7)	574 (75.4)	811 (66.3)	<0.001

* *p* values for linear trends across quartiles (assigned ordinal numbers 0–3) of green tea consumption are based on linear regression analysis for continuous variables and the Mantel test for categorical variables. ^†^ Values are mean (standard deviation) for continuous variables and number (percentage) for categorical variables.

**Table 3:** Multivariate-adjusted odds ratios (OR) and 95% confidence intervals (CI) of comprehensive frailty risk according to green tea consumption.

	Green Tea Consumption				*p* for Trend *	*p* for Interaction ^†^
	**None**	**<1 Cup/Day**	**1–2 Cups/Day**	**≥3 Cups/Day**		
Men, n	455	850	699	722		
No. of frailty (%)	136 (30.6)	255 (30.0)	222 (31.8)	197 (25.5)		
OR (95% CI)	1.00 (ref)	0.96 (0.74–1.25) ^‡^	1.07 (0.82–1.41)	0.71 (0.54–0.94)	0.02	
<75 years, *n*	317	599	436	444		0.01
No. of frailty (%)	71 (22.4)	138 (23.0)	110 (27.0)	88 (21.6)		
OR (95% CI)	1.00 (ref)	1.06 (0.76–1.48)	1.24 (0.87–1.76)	0.87 (0.60–1.25)	0.57	
≥75 years, *n*	127	250	110	107		
No. of frailty (%)	63 (49.2)	112 (44.6)	111 (42.5)	107 (32.6)		
OR (95% CI)	1.00 (ref)	0.78 (0.49–1.24)	0.77 (0.49–1.22)	0.46 (0.30–0.72)	<0.01	
Women, *n*	282	625	763	1232		
No. of frailty (%)	127 (45.0)	205 (32.8)	193 (25.3)	364 (29.6)		
OR (95% CI)	1.00 (ref)	0.67 (0.49–0.92)	0.51 (0.37–0.70)	0.60 (0.44–0.81)	<0.01	
<75 years, *n*	317	598	436	444		0.15
No. of frailty (%)	57 (33.1)	98 (24.0)	76 (15.2)	138 (18.5)		
OR (95% CI)	1.00 (ref)	0.66 (0.44–0.99)	0.41 (0.27–0.62)	0.51 (0.35–0.75)	<0.01	
≥75 years, *n*	110	217	262	485		
No. of frailty (%)	68 (61.8)	104 (47.9)	114 (44.5)	220 (45.4)		
OR (95% CI)	1.00 (ref)	0.68 (0.41–1.13)	0.60 (0.37–0.995)	0.61 (0.38–0.97)	0.076	

KCL, Kihon Checklist. IADL, instrumental activities of daily living. * Based on multiple logistic regression analysis assigning ordinal numbers 0–3 to quartile categories of protein intake. ^†^ Multiplicative interactions between green tea consumption and age groups. ^‡^ Adjusted for age (years), body mass index (kg/m^2^), total energy intake (kcal/day), alcohol status (everyday, sometimes, seldom, or never), smoking status (everyday, sometimes, former, or never), vegetable intake, fruit intake, and coffee consumption (almost none, <1 cup/day, 1–2 cups/day, ≥3 cups/day).

## Data Availability

The raw data are not publicly available.
